# Dasatinib-Triggered Severe Hypocalcemia in a Patient with Chronic Kidney Disease and Osteoporosis

**DOI:** 10.7759/cureus.48555

**Published:** 2023-11-09

**Authors:** Kohei Shiroshita, Mikio Okayama, Yosuke Oshima, Shinichiro Okamoto, Ken Sadahira

**Affiliations:** 1 Division of Hematology, Kawasaki Municipal Kawasaki Hospital, Kawasaki, JPN; 2 Division of Diabetes and Endocrinology, Kawasaki Municipal Kawasaki Hospital, Kawasaki, JPN

**Keywords:** hypocalcemia, long qt syndrome, osteoporosis, chronic kidney disease, dasatinib, chronic myeloid leukemia

## Abstract

Given the increasing prevalence of chronic myeloid leukemia (CML) in older individuals, careful selection of tyrosine kinase inhibitors (TKIs) is required. The case of an 84-year-old woman with chronic-phase CML and chronic kidney disease undergoing osteoporosis, in whom dasatinib triggered severe hypocalcemia, is reported. She was intolerant to both imatinib and nilotinib. Initiation of low-dose dasatinib treatment led to severe hypocalcemia and long QT syndrome, compounded by vitamin D deficiency and denosumab use. We stopped dasatinib, and her hypocalcemia was improved after calcium administration. This case highlights the potential of TKIs in triggering hypocalcemia, emphasizing the need to assess mineral disorders before initiating TKI therapy.

## Introduction

Tyrosine kinase inhibitors (TKIs) dramatically improve the prognosis of patients with chronic myeloid leukemia (CML) [1]. The incidence of CML increases with age and 20% of newly diagnosed patients with CML are over 70 years old in Japan [2]. The choice of TKIs in this population is crucial for successful treatment because older individuals have a multitude of comorbidities, such as hypertension (HT), diabetes mellitus (DM), dyslipidemia (DL), and chronic kidney disease (CKD). Furthermore, electrolyte abnormalities such as hypocalcemia should also be closely monitored during treatment with second-generation TKIs to avoid long QT syndrome.

CKD-mineral and bone disorders (CKD-MBD) impair calcium and phosphate metabolism. Vitamin D (vitD) is converted in the liver to 25-hydroxyvitamin D (25-(OH) vitD) and in the renal proximal tubule to 1α,25-dihydroxyvitamin D (1α,25-(OH) vitD). The parathyroid hormone (PTH), hypocalcemia, hypophosphatemia, and calcitonin activate 1α hydroxylase; in contrast, fibroblast growth factor 23 (FGF23) and 1α,25-(OH) vitD inhibit it due to CKD-related renal tubule damage. On the other hand, osteoporosis is characterized by low bone mass, microarchitectural deterioration of the bone tissue, and increased fracture risk. Bone remodeling involves osteoblasts and osteoclasts and is regulated locally and systemically [3]. Osteoclast differentiation is regulated by cytokines such as the receptor activator of nuclear factor-κB ligand (RANKL) and macrophage colony-stimulating factor (M-CSF). Furthermore, postmenopausal estrogen decline increases the risk of osteoporosis by increasing osteoclast RANKL. Therefore, osteoporosis treatment aims to prevent fractures by using bisphosphonates and RANKL inhibitors (RANKLi) [4].

Herein, we report the case of an 84-year-old female with chronic-phase CML (CML-CP) and CKD who was treated with TKIs combined with osteoporosis therapy and developed severe hypocalcemia and long QT syndrome after switching from TKI to low-dose dasatinib.

## Case presentation

An 84-year-old woman was admitted to our hospital for evaluation of leukocytosis in July 2022. She was being followed up for CKD, dyslipidemia, spinal stenosis, and osteoporosis, and her medical history was remarkable due to percutaneous catheter intervention for angina pectoris. She was administered aspirin, ezetimibe, and lansoprazole to treat these comorbidities. In addition, 60 mg of denosumab was also administered every 6 months (April and October) to treat osteoporosis 4 years before admission to our hospital. Physical examination findings were unremarkable. Peripheral blood showed leukocytosis, with an increased percentage of basophils and immature myeloid cells, including blasts, and high platelet counts. BCR-ABL1 mRNA in the peripheral blood was positive, while bone marrow examination revealed no increase in blasts. A karyotype analysis of bone marrow cells showed 46, XX, t(9;22)(q34;q11) in 20/20 metaphases. Based on these findings, the patient was diagnosed with chronic-phase CML (CML-CP) with a high Sokal score.

**Figure 1:**
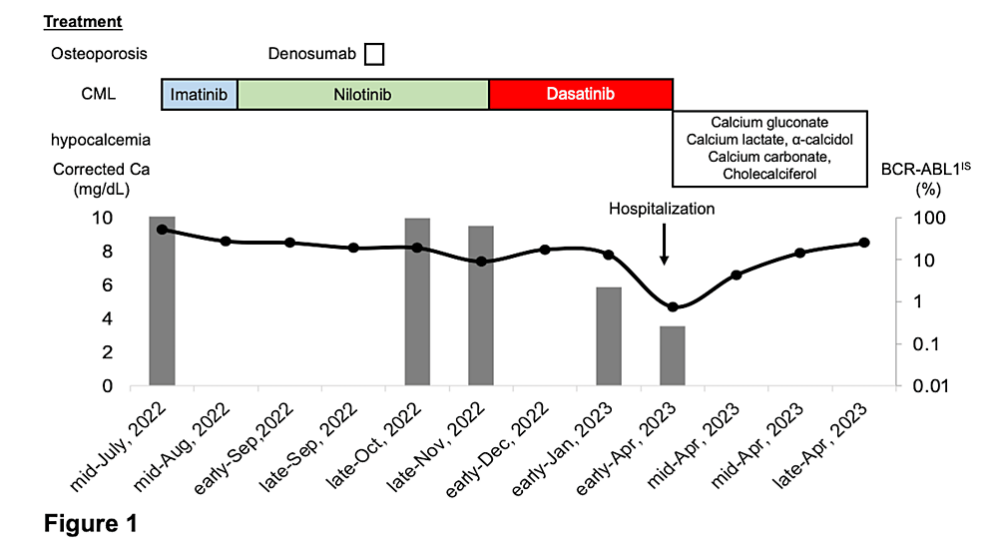
The clinical course in relation to serum calcium level and BCR-ABL1 value Because the patient was intolerant to imatinib and nilotinib, dasatinib was administered in October 2022. Since then, the BCR-ABL1^IS^ value steadily decreased and an optimal response was achieved; however, she presented with severe hypocalcemia 6 months after commencing dasatinib.

The clinical course of the patient is summarized in Figure 1. In mid-July 2022, the patient was administered 100 mg of imatinib per day, and the dose was gradually increased to 200 mg/day. However, imatinib treatment was discontinued due to worsening renal impairment. We switched to 150 mg nilotinib in mid-August; however, it was discontinued because of increased fatigue, reduced appetite, and severe anemia due to myelosuppression. Based on the results of the DAVLEC study [5], 20 mg of dasatinib was initiated. By January 2023 (3 months after switching to dasatinib), her BCR-ABL1 international scale (BCR-ABL1^IS^) decreased to 2.25% and achieved an optimal response based on European Leukemia Net 2020 criteria. In another clinic, 60 mg of denosumab was administered in early October; however, her serum calcium level was marginally below the normal range and remained slightly low till late October.

**Figure 2:**
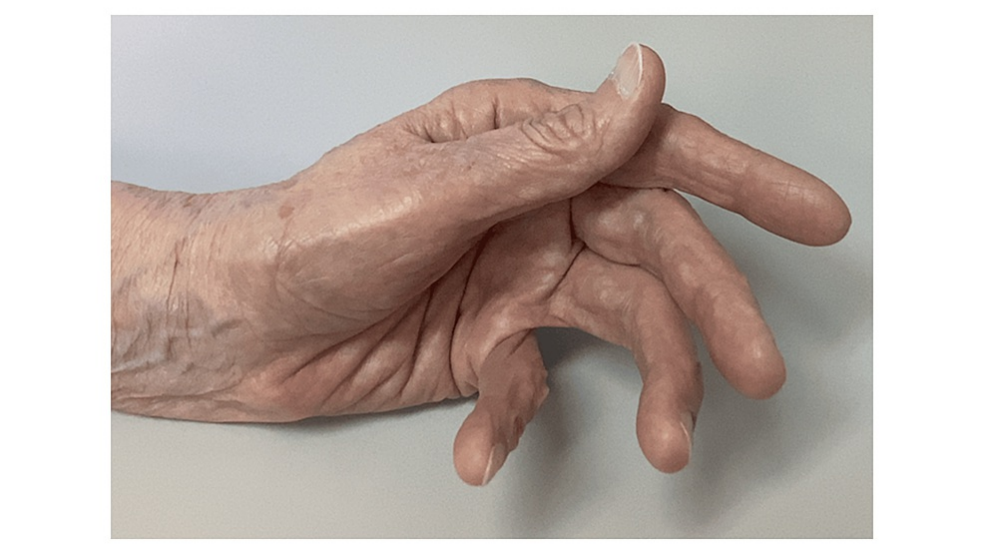
Trousseau sign after inflating the cuff for 3 minutes

In April 2023 (six months after switching to dasatinib), BCR-ABL1^IS^ dropped to 0.26%. However, she complained of fatigue and muscular weakness in both the upper and lower limbs. There were no episodes of loss of appetite or symptoms resembling a cold. Physical examination revealed a positive Trousseau sign (Figure 2).

The results of blood tests on admission are summarized in Table 1. Laboratory data showed hypocalcemia, hypophosphatemia, hypomagnesia, and increased creatinine levels. The intact PTH level was elevated. While 25-(OH) vitD remained undetectable, the level of 1α,25-(OH) vitD was normal.

**Table 1:** Laboratory tests

Test (Units of measurement)	Results	Reference range
White blood count (/μL)	4620	3500-9100
Neutrophil (%)	58.7	37-71
Lymphocyte (%)	29	20-50
Monocyte (%)	10.4	3-9
Eosinophil (%)	1.7	0-8
Basophil (%)	0.2	0-2
Red blood cell (×10^4^/μL)	262	380-480
Hemoglobin (g/dL)	8.7	11.3-15.2
Platelets (×10^4^/μL)	5.6	13.0-36.9
Total protein (mg/dL)	6.4	6.1-8.1
Albumin (mg/dL)	4	4.1-5.1
Total bilirubin (mg/dL)	0.4	0.2-1.2
Direct bilirubin (mg/dL)	0.2	0.0-0.3
Aspartate aminotransferase	21	10-35
Alanine aminotransferase	236	5-40
Lactate dehydrogenase (IU/L)	367	124-222
γ-Glutamyl transpeptidase (IU/L)	13	38-113
Blood urea nitrogen (mg/dL)	28	8-20
Creatinine (mg/dL)	1.92	0.2-0.80
Uleic acid (mg/dL)	3.6	2.4-5.6
Sodium (mEq/L)	143	135-146
Potassium (mEq/L)	4.6	3.4-4.8
Chlorine (mEq/L)	115	98-108
Calcium (mg/dL)	4.7	8.3-10.3
Phosphate (mg/dL)	2.3	2.4-4.5
Magnesium (mg/dL)	1.5	1.8-2.6
C-reactive protein (mg/dL)	0.27	0.0-0.3
Intact parathyroid hormone (pg/mL)	430	10-65
25-hydroxyvitamin vitamin D (ng/mL)	<30	>30
1,25-dihydroxyvitamin vitamin D (pg/mL)	26	20-60

**Figure 3:**
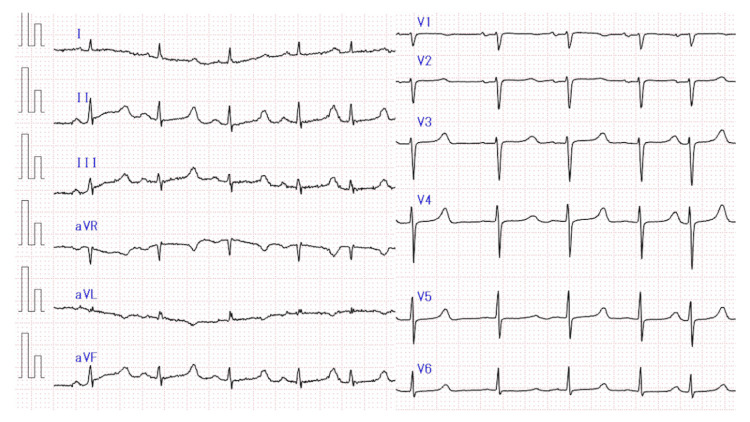
Electrocardiogram showing a prolonged QT interval

Furthermore, electrocardiography (ECG) revealed a heart rate of 95 beats/min, QRS was 72 ms, and the corrected QT (QTc) interval was prolonged to 503 ms, as calculated using the Bazett correction formula (Figure 3).

On the basis of these findings, the patient was diagnosed with long QT syndrome caused by severe hypocalcemia and secondary hyperparathyroidism due to vitamin D deficiency. Furthermore, her calcium levels were almost stable during imatinib and nilotinib, we also clinically determined that dasatinib triggered hypocalcemia.

Therefore, we discontinued dasatinib treatment and immediately initiated intravenous calcium replacement with cholecalciferol and magnesium carbonate. Her calcium level was successfully returned to the normal range two weeks after treatment, and symptoms and long QT syndrome were completely resolved. Asciminib was then administered to treat the CML, and no recurrence of hypocalcemia was observed.

## Discussion

This case emphasizes the potential of TKIs to worsen bone and mineral imbalances in patients with CKD undergoing RANKLi treatment. The incidence of grade 3/4 hypocalcemia is reported as only in 3% in the DASISION trial [6] and 0% in the DAVLEC trial [5]. However, our case implies that dasatinib-induced hypocalcemia may be more frequently observed in clinical practice, especially in patients with vitD deficiency due to CKD or osteoporosis, who are also treated with RANKLi.

TKIs are widely used to treat CML and imatinib, a 1st generation TKI, has demonstrated long-term efficiency and safety [7]. In contrast, second-generation TKIs, including dasatinib, have been shown to achieve prompt and prolonged remission than imatinib [8]. However, these TKIs have distinct off-target effects owing to their specific kinase inhibition. Imatinib and nilotinib inhibit c-Kit and the platelet-derived growth factor receptor (PDGFR) and suppress vascular regeneration and angiogenesis. Moreover, imatinib causes renal failure and fluid overload [9], whereas nilotinib increases the risk of vascular occlusion [10]. Dasatinib broadly inhibits tyrosine kinases, including the Src family, c-Kit, and PDGFR. Its major adverse events include pulmonary HT and pleural effusion [8]. Recently, Japanese investigators reported the efficacy and safety of low-dose dasatinib induction [5], suggesting that a reduced TKI dose might be more tolerable and may decrease adverse events while maintaining efficacy in older patients with CML.

Furthermore, previous studies have reported that TKIs affect bone and mineral homeostasis. Hypophosphatemia, secondary hyperparathyroidism [11], and osteoporosis [12] are observed following imatinib treatment because imatinib inhibits PDGFR, which promotes osteoblast differentiation and suppresses osteoclast differentiation [13]. Nilotinib also suppresses PDGFR-dependent osteoblast proliferation and osteoclastogenesis by stromal-cell-dependent mechanism in vitro [14]. The inhibition of c-Src and PDGFR, which are off-targets of dasatinib, enhances osteoblast differentiation and bone formation and suppresses bone absorption [15-18]. However, the clinical effect of second-generation TKIs on calcium and phosphate metabolism in older patients with CKD-MBD and osteoporosis remains unclear.

vitD concentration declines earlier than the rise in serum PTH levels and hyperphosphatemia and tends to decrease with the progression of CKD stages, leading to decreased calcium absorption from the intestine and inhibition of phosphate excretion. Moreover, in preserved-stage CKD, there is evidence of a positive correlation between serum 1α,25-(OH) vitD concentration and creatinine clearance [19]. Denosumab undergoes degradation within the body, with a half-life of approximately 21 days. Therefore, most hypocalcemia associated with denosumab occurs within 7 days after administration, and a significant proportion occurs between 1 and 2 months post-administration. The incidence of hypocalcemia after denosumab administration was higher in patients with CKD [20].

Our patient did not have severe hypocalcemia at the diagnosis of CML-CP, suggesting that vitD deficiency due to CKD-MBD was subclinical at the time of CML-CP. Furthermore, denosumab was administered for 4 years, yet serum calcium levels remained within normal limits, and there were no significant symptoms associated with hypocalcemia. Notably, severe hypocalcemia occurred immediately prior to subsequent doses. This suggests that denosumab may not be the sole definitive cause based on the duration of its half-life, although RANKLi likely contributes to severe hypocalcemia. Moreover, hypocalcemia progressed slightly following denosumab administration during nilotinib treatment. This could be explained by the reduced calcium intake resulting from decreased appetite due to nilotinib and denosumab administration. The slight but insignificant recovery in calcium concentration observed upon discontinuation of nilotinib may support this speculation.

Based on these findings, we hypothesized that dasatinib may play a significant role in triggering severe hypocalcemia in the context of CKD-MBD and ongoing denosumab therapy. In patients with CKD, vitD administration reportedly improves prognosis. In addition, supplementation with calcium and vitD is recommended during denosumab treatment; however, our patient did not receive these supplements. Therefore, we should have checked the risk of vitD deficiency due to CKD-MBD and history of therapy for osteoporosis. When patients have these comorbidities, optimal supplementation to increase serum calcium levels should be initiated before initiating TKI treatment.

## Conclusions

We present a case of CML with CKD in a patient on medication for osteoporosis who experienced severe hypocalcemia and long QT syndrome soon after initiating low-dose dasatinib. We speculate that multiple factors, including vitD deficiency, RANKLi, hypomagnesemia and dasatinib, contributed to the severe hypocalcemia observed in this case. Furthermore, careful evaluation of drug interactions is required to avoid complications because older individuals are likely to have multiple comorbidities. Therefore, clinicians should consider bone and mineral imbalances before initiating TKIs in patients with CKD undergoing osteoporosis therapy.
